# Physical exercise programmes to improve insomnia or poor sleep quality in non-hospitalised elderly people: a systematic review and meta-analysis

**DOI:** 10.7717/peerj.20764

**Published:** 2026-02-16

**Authors:** Laura Pilar De Paz-Montón, Juan Manuel Carmona-Torres, Ángel López-Fernández-Roldán, Rosa María Molina-Madueño, Carlos Navarrete-Tejero, José Alberto Laredo-Aguilera

**Affiliations:** 1 Faculty of Physiotherapy and Nursing, University of Castilla-La Mancha, Toledo, Spain; 2 Multidisciplinary Research Group in Care (IMCU), University of Castilla-La Mancha, Toledo, Spain; 3 Institute of Health Research of Castilla-La Mancha (IDISCAM), Toledo, Spain

**Keywords:** Physical therapy modalities, Exercise, Sleep, Actigraphy, Polysomnography

## Abstract

**Background:**

Insomnia, or poor quality of sleep, among older people increasingly affects both physical and psychological health. This study aimed to evaluate the efficacy of physical exercise programs for improving sleep quality in non-hospitalized older adults, *via* objective methods such as actigraphy and polysomnography.

**Methods:**

A systematic review and meta-analysis was conducted between January 1, 2025 and March 31, 2025 according to the standards of the Preferred Reporting Items for Systematic Reviews and Meta-Analyses (PRISMA) guide. It has been registered in Prospero with the number CRD420251009838. The following databases were consulted: PubMed, Web of Science, CINAHL Complete and Scopus. The studies included groups of participants with a mean age above 60 years, who presented with sleep problems, insomnia or an interest in improving sleep quality. To assess the quality of the studies, the Rob-2 tool for randomized studies and crossover studies and the critical appraisal of the Joanna Briggs Institute were used.

**Results:**

Seven experimental or quasi-experimental studies with intervention groups and/or controls published in the last decade were analyzed. Interventions included aerobic exercise, resistance training, tai chi, and aquatic activities. The results demonstrated improvements in sleep latency, sleep efficiency, total sleep time, and wake time after sleep onset. Meta-analyses confirmed statistically significant benefits, particularly in terms of sleep latency and efficiency.

**Conclusions:**

The findings support the implementation of physical activity programs as cost-effective, safe, and practical interventions to enhance sleep quality in community and healthcare settings.

## Introduction

From an epidemiological perspective, progressive population aging is observed, with a predominance of industrialized countries. Globally, between 2025 and 2050, the proportion of people over 60 years of age is projected to double from 12% to 22%. Furthermore, in 2023, the life expectancy at birth in the European Union was 81.5 years (Conde-Ruiz & González, 2021; National Statistics Institute (INE), 2022). Similarly, there is a significant difference between the sexes since women have a life expectancy of 84 years and men have a life expectancy of 78.5 years, indicating that women constitute 55% of the population over 65 years of age and 62% of the elderly population over 80 years old (Conde-Ruiz & González, 2021; National Statistics Institute (INE), 2022; United Nations, Department of Economic and Social Affairs, 2020). In recent years, those aged 60 years and over have been recognized as older adults according to international organizations (World Health Organization, 2022; United Nations, Department of Economic and Social Affairs, 2017), and this is the cut-off point in various studies (Porcel-Gálvez et al., 2021; Zhang et al., 2024) since it is the period in which bodily functions can begin to experience certain decreases or the appearance of pathologies.

Aging is the multidimensional process resulting from the cellular and molecular damage that occurs over time (World Health Organization, 2023). This aging entails progressive physical, psychological and mental changes, and there is an increased risk of disease (Piña Morán et al., 2022). In addition, it is usually accompanied by important vital and social transitions, such as retirement, the loss of loved ones or the need for adjustments at home to maintain autonomy (World Health Organization, 2023; Piña Morán et al., 2022). This moment represents a key opportunity to promote active aging, healthy habits, physical activity, emotional well-being and disease prevention, with the aim of maintaining autonomy and quality of life in later stages (ASISTED Blog, 2019).

However, despite these preventive strategies, in old individuals, the appearance of chronic pathologies and multifactorial conditions known as geriatric syndromes is common, which significantly impacts the functionality and autonomy of the elderly (Schwab, 2008), highlighting falls, cognitive impairment and/or delirium; polypharmacy, which is an extremely common condition among the elderly with the consequent economic expense and problems for the metabolism that it entails; functional limitations and mobility impairments, which hinder the performance of basic and instrumental activities of daily living (Cesari et al., 2016); and sleep disorders or poor quality of sleep, which are factors that increase the risk of cognitive and physical deterioration if not timely (Cesari et al., 2016; Cruz et al., 2018).

Within the framework of geriatric syndromes, sleep disorders are often conceptualized as secondary or interrelated conditions, rather than as isolated primary outcomes. They frequently coexist with other syndromes, such as cognitive decline, frailty, and depressive symptoms, and contribute to their progression. Despite their secondary classification, sleep disorders exert a significantly influence the functionality, autonomy, and overall quality of life of older adults, underscoring their clinical relevance and the need for specific non-pharmacological interventions (Cesari et al., 2016; Morley, 2017).

Currently, active or healthy aging is promoted, a term developed in the 1990s as the possibility of aging in the absence of chronic diseases and without physical disabilities, continuing with participation in society and including social and mental well-being (Paul, Ribeiro & Teixeira, 2013; Abud et al., 2022). In addition, determinants of healthy aging, including a good diet, lifelong learning, physical activity, self-knowledge, social support, faith, independence, good quality of sleep and community participation, have been defined (Abud et al., 2022; Petretto et al., 2016). To promote this process, societies and governments must adapt public environments and policies to meet the needs of older adults. In this context, the World Health Organization (WHO) promoted a global strategy between 2016 and 2020 (Paul, Ribeiro & Teixeira, 2013) and is currently leading the Decade of Healthy Aging (2020–2030). This initiative prioritizes research, the fight against ageism and the adaptation of health systems. All of these efforts seek to improve the quality of life of the elderly population (Paul, Ribeiro & Teixeira, 2013; Rudnicka et al., 2020).

As mentioned above, poor quality of sleep or insomnia is one of the main problems that affects elderly individuals, since with aging, there is a decrease in the circadian rhythm, affecting the duration, schedule and consolidation of sleep (Mattis & Sehgal, 2016), indicating that this fragmentation is associated with earlier cognitive deterioration and memory problems than those who do not suffer from this problem (Mander et al., 2017). In addition, poor sleep causes emotional and physical problems and is even associated with increased mortality and morbidity (Chaput et al., 2020). Worldwide, evidence indicates that between 30% and 48% of older adults suffer from insomnia symptoms (Pradhan & Saikia, 2024), which affect women to a greater extent, as a result of hormonal changes, body temperature or melatonin (Kravitz & Joffe, 2018; Carmona Fortuño & Molés Julio, 2018).

The most common form of treatment is pharmacological, despite being considered a complementary treatment if other types of therapies are not effective (Abad & Guilleminault, 2018; Patel, Steinberg & Patel, 2018). In this sense, the use of benzodiazepines, antidepressants, antipsychotics and melatonin as pharmacological sleep treatments stands out. However, different therapies are currently being studied with the aim of reducing the drug burden and opting for treatments that are safer, easy to implement and plausible in terms of cost effectiveness for health systems. Among them are aromatherapy (Her & Cho, 2021), cognitive interventions, (Lovato et al., 2014; Alessi et al., 2016; Lovato, Lack & Kennaway, 2016) education about sleep hygiene, (Patel, Steinberg & Patel, 2018) and physical activity programs, which have shown positive results (Lin et al., 2020).

Physical activity, in particular, has been prioritized among non-pharmacological interventions because it exerts multidimensional benefits on sleep physiology and overall health. Unlike other approaches such as cognitive behavioral therapy, aromatherapy, or sleep hygiene education, which primarily act through psychological or sensory pathways, exercise directly influences core regulatory mechanisms of sleep, including thermoregulation, circadian rhythm alignment, and neuroendocrine balance (*e.g*., melatonin, cortisol and serotonin). Furthermore, it enhances cardiovascular fitness, cognitive performance, and mood, all of which are closely interrelated with sleep quality in older adults. Its feasibility, safety, and cost-effectiveness make it an ideal therapeutic alternative for insomnia and an optimal candidate for large-scale implementation in both community and clinical contexts (Kredlow et al., 2015; Passos et al., 2011; Yu et al., 2025).

Although systematic reviews have been carried out on the benefits of physical activity on sleep (De Nys et al., 2022; Memon et al., 2021; Saunders et al., 2016; Alnawwar et al., 2023), to our knowledge, there have been no systematic reviews that analyze the impact of physical activity programs on the treatment of insomnia and improvement in the quality of sleep in elderly individuals *via* objective measurements such as polysomnography or actigraphy. The use of objective measurements, such as polysomnography and actigraphy, is essential to accurately assess the effects of interventions on sleep, as they provide a more accurate and reliable assessment than subjective questionnaires do, reducing bias and improving the validity of results obtained in sleep studies (Haghayegh et al., 2019; Neikrug, 2023; Fekedulegn et al., 2020). These instruments capture subtle physiological changes undetectable through subjective questionnaires, ensuring greater methodological rigor in intervention studies. Nevertheless, their application in large-scale or community settings may face feasibility challenges, including higher cost, the need for technical expertise, and lower participant tolerance. Balancing these aspects, the present review focuses on studies that implement objective tools while recognizing that combining them with validated subjective measures can provide a more comprehensive evaluation of sleep outcomes (Haghayegh et al., 2019; Ancoli-Israel & Martin, 2023).

The main objective of this study was to objectively evaluate the impact of different physical activity programs on sleep in non-hospitalized people over 60 years of age. As a secondary objective, sleep measured by subjective methods will also be evaluated.

## Materials and Methods

### Design and sources of information

A systematic review was carried out following the guidelines of the Preferred Reporting Items for Systematic Reviews and Meta-Analyses (PRISMA) statement (Page et al., 2020).

For this purpose, a search was carried out in the following databases: PubMed, Web of Science, CINAHL complete and Scopus. The search was last updated on 15 March 2025.

This systematic review has been registered in Prospero with the number CRD420251009838 (De Paz-Montón, Carmona-Torres & Laredo-Aguilera, 2025).

### Search strategy

The research was carried out following the PICO question shown in Table 1. The search strategy is shown in Table 2.

**Table 1:** Research questions in the PICO format.

Population (P)	Intervention (I)	Comparator (C)	Results (O)
People over 60 years of age with insomnia, poor quality of sleep and/ or willingness to improve the quality of sleep.	Physical activity program.	People over 60 years of age to whom no physical exercise intervention is applied.	Efficacy and quality of sleep measured with actigraphy or polysomnography and subjective methods.

**Note:**

Own elaboration.

**Table 2:** Search strategy used for each database.

Database	Search strategy
PubMed	((physical activity [Title/Abstract]) AND (Aged [Title/Abstract] OR “older people” [Title/Abstract] OR elderly [Title/Abstract])) AND (sleep [Title/Abstract]))
CINAHL complete	
Web of Science	(physical activity AND (Aged OR “older people” OR elderly) AND sleep AND actigraphy)

**Note:**

Own elaboration.

### Inclusion and exclusion criteria

The inclusion criteria were as follows: (1) Experimental or quasi-experimental studies with a control group. (2) People with an average age greater than 60 years with sleep problems, insomnia or a disposition to improve the quality of sleep. (3) Studies on physical activity interventions designed to treat sleep problems and evaluate improvements in sleep-related variables. (4) Studies in which objective sleep assessment tools are used.

The exclusion criteria were as follows: (1) Studies older than 10 years. (2) Older adults who are hospitalized or have serious pathologies. (3) Studies published in languages other than English.

We included studies published from January 2015 to March 2025 to ensure the scientific quality and update of the articles (Furuya-Kanamori et al., 2023).

We defined older adults as those aged 60 years or older. This threshold is consistent with the definition used by the World Health Organization in its active aging framework, which uses 60 years as the starting point for old age in many global contexts. Furthermore, recent studies analyzing the operational definition of old age revealed that, while thresholds vary (40–77 years), the most frequent cutoffs in epidemiological studies are 60 years (20.4%) and 64 years. This suggests that ≥60 years is a credible and frequently used criterion. Using ≥60 years allows us to include the early phase of aging, when changes related to sleep, circadian rhythm, and activity begin to emerge, thus expanding the relevance of our review to interventions in community-dwelling older adults (Truijen et al., 2025; World Health Organization, 2002; Petrovsky et al., 2021).

### Selection process

The study selection process was carried out collaboratively between Laura Pilar de Paz-Montón (LPPM) and Juan Manuel Carmona Torres (JMCT), following the established inclusion and exclusion criteria.

Citations were organized, and duplicates were eliminated, *via* the Mendeley Reference Manager. After duplicates were removed, titles and abstracts were reviewed to identify studies requiring further examination.

After a full-text analysis, the final selection of the studies to be included in the qualitative and quantitative synthesis was carried out. In cases where discrepancies arose during the selection process, discussions were held between LPPM and JMCT, and, if consensus was not reached, a third author, José Alberto Laredo Aguilera (JALA), was consulted to resolve disagreements and reach a consensus.

### Evaluation of the quality of the studies

The methodological quality of the included studies was assessed *via* validated risk-of-bias tools according to the study design. For randomized controlled trials, we applied the Revised Cochrane Risk-of-Bias Tool (RoB-2) (Sterne et al., 2019), which evaluates five domains: randomization process, deviations from intended interventions, missing outcome data, outcome measurement, and selection of the reported results. For crossover trials, we used the adapted RoB-2 for crossover designs, which additionally considers period and carry-over effects (United Nations, Department of Economic and Social Affairs, 2017).

The studies were considered of sufficient quality when at least 4 of the 5 items marked “low risk” and only one of them could indicate “some concern”. This approach ensured consistency across designs while maintaining methodological rigor (Sterne et al., 2019; Higgins et al., 2021). For quasi-experimental or pre–post studies without randomization, we applied the Joanna Briggs Institute (JBI) Critical Appraisal Checklist for Quasi-Experimental Studies (Joanna Briggs Institute, 2020). The studies were considered to have sufficient quality when they obtained at least 7 “yes” out of the nine possible answers on the basis of previous studies (Barker et al., 2024).

### Extraction of the results

The extraction of the results was carried out *via* LPPM and JMCT. The results were extracted *via* Mendeley Reference Manager, classified manually and comprehensively analyzed, extracting the following information from each article: first author, year and country; study design; characteristics of the participants, sample size, mean age, sex and selection; study intervention; main results (latency period, sleep efficiency, total sleep time and wake-up time after starting sleep),; measurement methods and evaluations (actigraphy, polysomnography, questionnaires, sleep diary, *etc*.); and losses and limitations.

### Analysis of the data obtained

A qualitative synthesis of each study included in this review was performed. The influence of physical activity interventions on the effectiveness and quality of sleep was analyzed, both objectively and subjectively, before and after the intervention.

For the quantitative synthesis, random-effects meta-analyses were performed *via* RevMan 5.4 to evaluate the impact of physical activity interventions on sleep quality.

Statistical heterogeneity was assessed *via* the I^2^ statistic and was defined as follows: intervals of I^2^ ≤ 25%, 26–50%, and ≥51% indicated low, medium, or high heterogeneity, respectively and statistical significance was set at 0.05.

## Results

### Selection and characteristics of the studies

**Figure 1:**
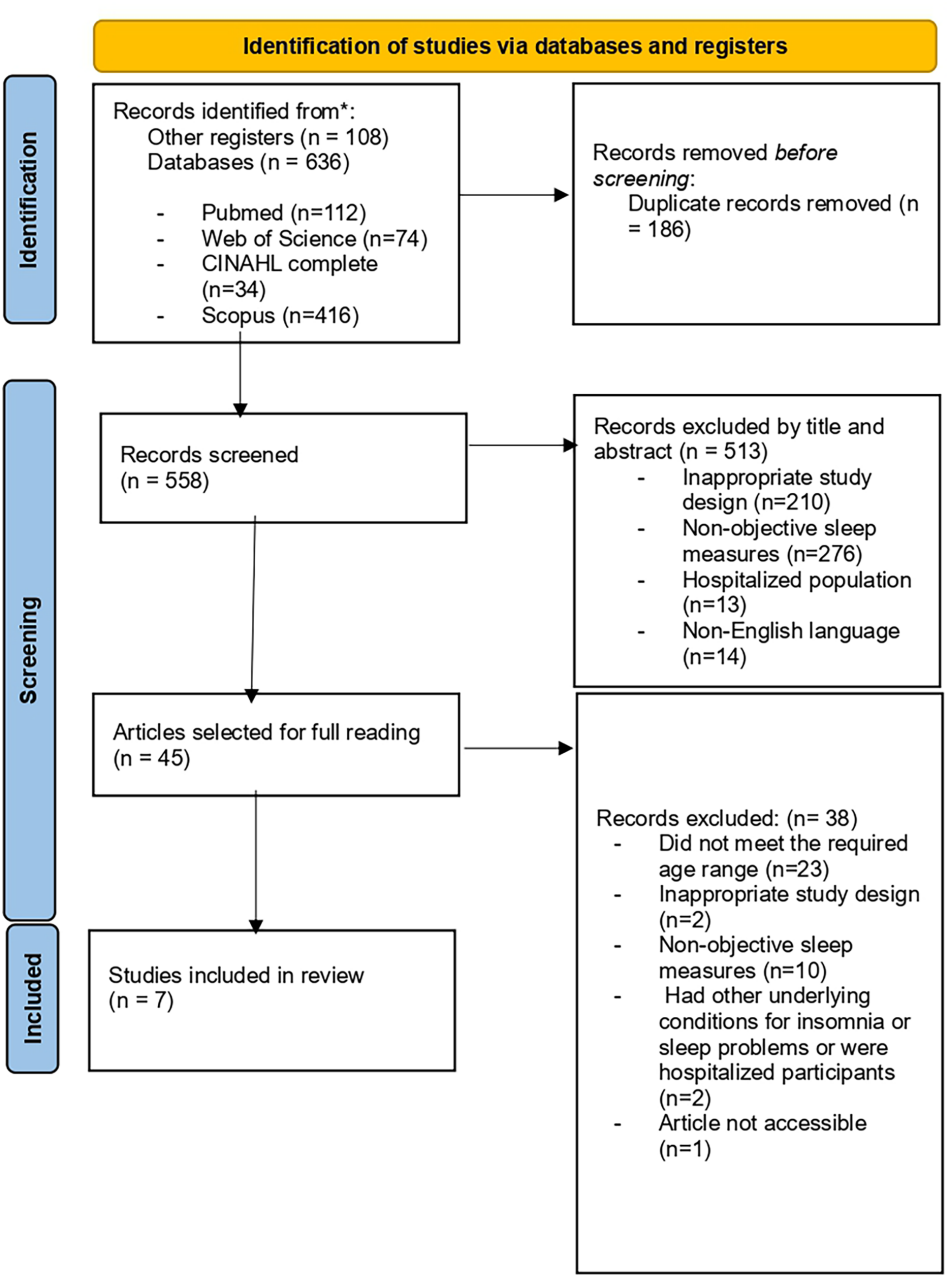
Flowchart for the selection of studies. Study selection according to systematic review process.

Through searches of the different databases and registries, a total of 744 studies were identified. Later, 186 duplicates were eliminated, leaving 558 studies. After the titles and abstracts were read, only 45 were selected for full-text reading and subsequent analysis. Finally, a total of seven studies were included. Figure 1 shows the PRISMA diagram for the selection of articles in systematic reviews. The studies cited a total of 984 participants, of which 684 were women. Four of the included studies were randomized clinical trials with a control group (Chen et al., 2016, 2019; Tseng et al., 2020; Siu et al., 2021), one was a randomized clinical trial without a control group where it was compared with another intervention (Seol et al., 2021a), one was a 3-way crossover study (Seol et al., 2021b), and one was a pre-test post-test clinical study (Vanderlinden et al., 2022).

All studies included participants with a mean age over 60 years with sleep problems, insomnia and/or a willingness to improve the quality of sleep.

Each intervention met the established ethical criteria, and favorable reports were obtained from the corresponding ethics committees.

Table 3 presents the data analysis and main characteristics of the studies included in this systematic review.

**Table 3:** Main characteristics of the studies included in the systematic review.

First author, year and country	Design	Participants and characteristics	Interventions	Evaluation and results	Conclusions
Chen et al. (2016). Taiwan.	RCT	*N* = 63	IG: 8-week aquatic exercise program with 2 weekly sessions of 60 min.	Actigraphy parameters and subjective measurements.	The IG has significant improvements in latency and efficiency over CG.
		IG: 33 (22 W)	CG: no intervention. It was recommended to continue with your usual daily activity.	T0: pre intervention.	In addition, daytime naps were not taken into account.
		CG: 34 (28 W)		T1: post intervention.	
		A: 65,7		Sleep latency: improvement in IG of −1,3 min. *p* = 0.011.	
		C: (1) No work, no cognitive impairment. (2) With poor subjective sleep quality. (3) Excluded if diagnosed with a sleep disorder or any condition that could limit their participation in moderate intensity exercises.		Sleep efficiency: improvement in IG compared to CG. *p* < 0.001.	
				TTS: no significant differences in IG with respect to CG.	
				WASO: no significant differences.	
Chen et al. (2019). Taiwan.	RCT	*N* = 40	IG: 1 supervised session of light walking of 50 min on a treadmill.	Actigraphy parameters.	There are significant improvements in IG in latency and sleep efficiency relative to CG.
		IG: 20 (M)	CG: I rest in a waiting room for 2 h, where they had books, magazines and water.	T0: 2 days before. intervention.	Only women have been included.
		CG: 20 (M)		T1: 2 days post intervention.	
		A: 60,4		Sleep latency: improvement in IG de −3,3 min. *p* < 0.001.	
		C: (1) women (2) no cognitive impairment. (3) no regular exercise.		Sleep efficiency: improvement in IG compared to CG. *p* = 0.025.	
				TTS: no significant differences in IG with respect to CG.	
				WASO: no significant differences.	
Tseng et al. (2020). Taiwan.	RCT	*N* = 40	IG: 12-week program of physical exercise (moderate intensity aerobic exercises, resistance training and flexibility routines) with 3 weekly sessions of 50 min.	PSQI and actigraphy parameters.	There are significant improvements in sleep efficiency, sleep quality and a decrease in the latency period in IG compared to CG.
		IG: 20 (18 W)	CG: no intervention is applied.	T0: previous intervention.	Furthermore, most of the participants are women.
		CG: 20 (18 W)		T1: post intervention.	
		A: 62, 2		Sleep latency: −8,3 min improvement in IG. *p* = 0,004.	
		C: (1) PSQI > 5 (2) no regular physical exercise in the previous 3 months. (3) No uncontrolled psychiatric, sleep or cardiovascular pathologies.		Sleep efficiency: improvement in IG compared to CG. *p* = 0.088.	
				TTS: mejora en IG de 19.7 min. *p* = 0.271	
				WASO: no significant differences.	
Seol et al. (2021a). Japan.	Clinical trial: 3-way crossover study.	*N* = 10 (M)	IG1: 1 30-min step exercise session.	OSA-MA questionnaire and polysomnography.	IG1 and IG2 obtained significant improvements in sleep latency compared to CG.
		IG1: 10	IG2: 1 session where directed low intensity domestic tasks are performed.	T1: sleep wakefulness.	This is a small sample where only women are included.
		IG2: 10	CG: remain seated.	T2: non rapid eye movement.	
		CG: 10		T3: rapid eye movement.	
		A: 72,3		T4: upon awakening by questionnaire.	
		C: (1) No sleep medication. (2) No restriction of physical exercise or habit. (3) They do not smoke. (4) Bedtime between 21:00 and 23:00.		Sleep latency: significant improvement in IG1 and IG2. *p* = 0.011.	
				Sleep efficiency: improvement in IG1 and IG2 compared to CG. *p* = 0.902.	
				TTS: no significant differences.	
				WASO: no significant differences	
Siu et al. (2021). Honk Kong.	RCT	*N* = 320	IG1: 12-week program of physical activity (walking and muscle strengthening exercises) with 3 weekly sessions of 1 h per week.	PSQI, ISI, sleep diary and actigraphy.	Significant improvements have been obtained in both objective and subjective sleep parameters in IG1 and IG2, being very similar post intervention and remaining after 24 months.
		IG1: 105 (84 W)	IG2: 12-week Tai-chi program with 3 weekly sessions of 1 h.	T0: pre intervention.	The intervention was developed in a single center, which may limit its generality
		IG2:105 (84 M)	CG: no intervention.	T1: post intervention.	
		CG: 110 (88 M)		T2: 24 months post intervention.	
		A: 67,27		Sleep latency: significant improvement in IG1 and IG2 with respect to CG. *p* = 0.001.	
		C: (1) Insomnia according to DMS-5. (2) No regular physical exercise. (3) No other moderate pathologies. (4) No disability of physical activity.		Sleep efficiency: improvement in IG1 and IG2 compared to CG. *p* = 0.001.	
				TTS: mejora en IG1 e IG2. *p* = 0.71.	
				WASO: improvement in IG1 and IG2 compared to CG. *p* < 0.001.	
Seol et al. (2021b). Japan.	RCT no control group	*N* = 60	IG1: physical exercise program developed in the mornings.	Actigraphy, PSQI and sleep diary.	Low intensity exercise at home, particularly when practiced in the afternoon, is an effective non pharmacological method of improving sleep.
		IG1: 30 (22 W)	IG2: same physical exercise program performed in the afternoon.	T0: pre test	
		IG2: 30 (23 W)	The program runs for 8 weeks with 30 min. daily low intensity aerobic exercise.	T1: 4 weeks after starting the intervention	
		20 of each group have good quality of sleep and 10 poor.		T2: at 8 weeks post-test.	
		A: 71		Sleep latency: improvement in IG2 compared to IG1. *p* = 0.016.	
		C: (1) No sleep medication. (2) No exercise restriction.		Sleep efficiency: no significant differences between IG1 and IG2. *p* = 0.021.	
				TTS: mejora en IG1 respecto a IG2. *p* = 0.010.	
				WASO: mejora en IG1 respecto a IG2. *p* = 0.002.	
Vanderlinden et al. (2022). Belgium.	Clinical trial pretest post test	*N* = 451	IG: 12-week training program with a weekly session of 60 min.	PSQI and actigraphy.	In this case, there are no significant differences between IG and CG in sleep, meaning that a weekly session is not enough.
		IG:261 (145 W)	In addition, a 2-h session of healthy nutrition was held.	T0: pre test	
		CG:192 (102 W)	CG: no intervention is applied.	T1: post test	
		A: 71,75		Sleep latency: NA	
		C: (1) Willingness to improve your quality of sleep. (2) Be able to follow the training program.		Sleep efficiency, TTS, WASO: no statistically significant improvements.	

**Note:**

A, age; C, characteristics; CG, control group; IG, intervention group; Interv, intervention; ISI, Insomnia Severity Index; Min, minutes; OSA–MA, Oguri-Shirakawa-Azumi Sleep Inventory; PSQI, Pittsburgh Sleep Quality Questionnaire; TTS, total sleep time; WASO, awakenings after sleep onset; W, women.

### Risk of bias

The seven included studies were assessed for risk of bias *via* the critical appraisal of JBI and by RoB Checklist tools (Sterne et al., 2019; Higgins et al., 2021; Joanna Briggs Institute, 2020; Chen et al., 2016, 2019; Tseng et al., 2020; Siu et al., 2021; Seol et al., 2021a, 2021b; Vanderlinden et al., 2022).

**Figure 2:**
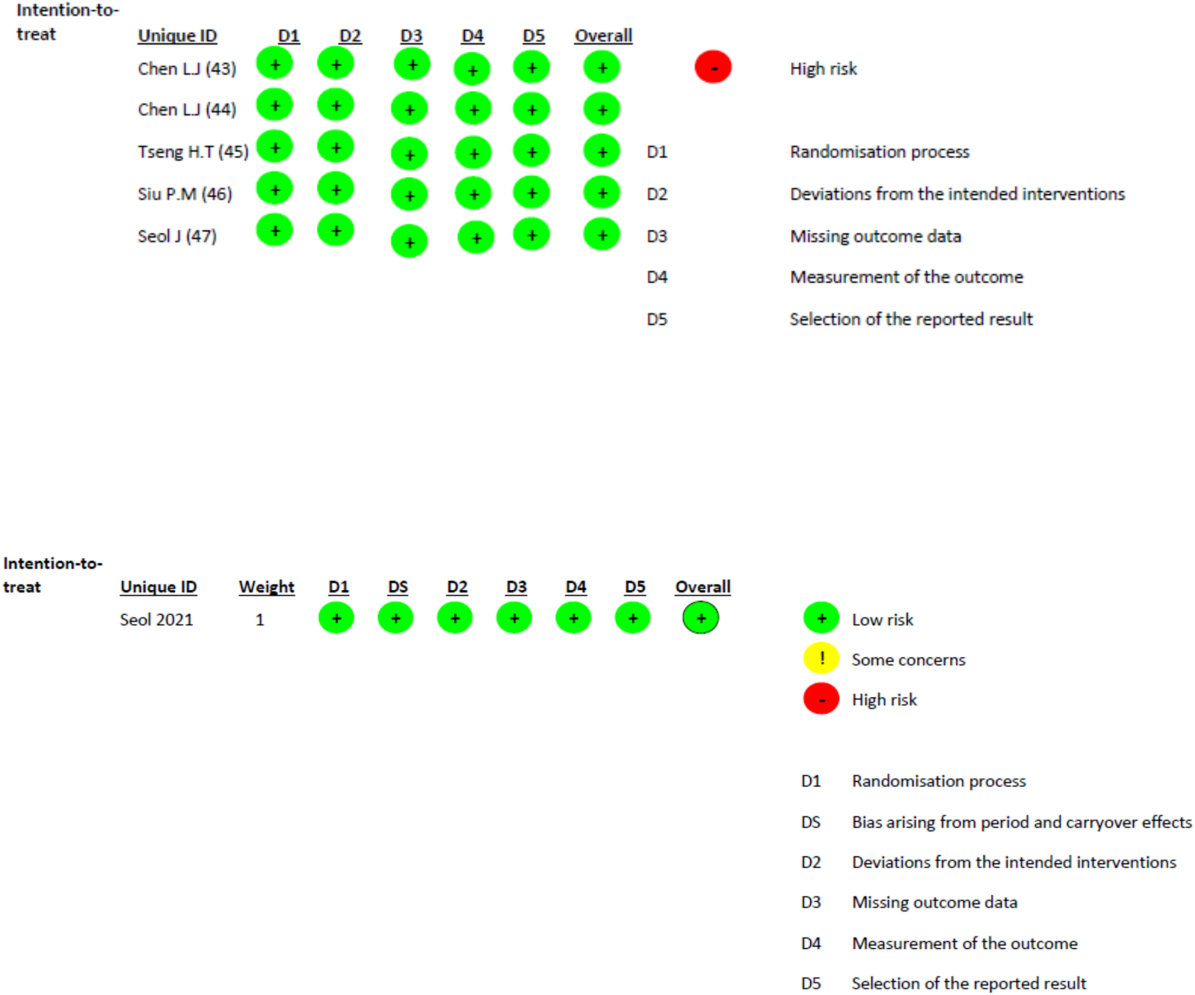
Application of the Rob-2 tool for randomized clinical trials. Domains: D1–Randomization process D2–Deviations from intended interventions D3–Missing outcome data D4–Measurement of the outcome D5–Selection of the reported result (and D5b–Bias arising from period or carry-over effects in crossover designs).

Figure 2 shows the evaluation of the different domains for the studies that were randomized clinical trials. Table S1 shows the evaluation of the JBI scale for the pretest post-test study.

According to the RoB-2 assessments (Sterne et al., 2019; Higgins et al., 2021) all included studies were rated as having a low risk of bias across the evaluated domains. For the case of the study by Vanderlinden et al. (2022), the Joanna Briggs Institute (JBI) Critical Appraisal Checklist for Quasi-Experimental Studies was used. All items received a “yes” response except for the one asking if the participants were included in any comparison receiving similar treatment care, other than the exposure or intervention of interest, which received a “no” response. Therefore, since eight out of nine items received “yes” responses, the risk of bias was low (Barker et al., 2024).

These outcomes reflected adequate randomization, appropriate handling of outcome data, and clear reporting of results in the selected trials. However, some methodological constraints inherent to exercise interventions, such as the impossibility of participant blinding and relatively small sample sizes, should be acknowledged when these findings are interpreted. Although these factors did not raise the overall risk classification, they may still influence the precision and generalizability of the pooled estimates.

### Physical activity programs

The seven studies included in this review investigated the effectiveness of physical activity programs in improving sleep in older adults. The interventions were diverse, and included aquatic exercise, walking, aerobic training, tai-chi, or muscle strengthening, resulting in significant improvements in sleep efficiency, a reduction in sleep-onset latency, and decreased nighttime awakening. In addition, subjective benefits were observed in the perception of the quality of sleep. However, studies with lower frequency of sessions or low adherence did not yield statistically significant results, which reinforces the importance of the design and consistency of the intervention over time (Vanderlinden et al., 2022).

### Objective methods for measuring the quality of sleep

The objective quality of sleep and its different parameters have been evaluated mainly by using actigraphy and polysomnography to objectively measure sleep parameters and average motor activity over a period of days to weeks. Actigraphy was used to measure the objective quality of sleep in six of the studies (Chen et al., 2016, 2019; Tseng et al., 2020; Siu et al., 2021; Seol et al., 2021a; Vanderlinden et al., 2022), whereas polysomnography was used in only one of them (Seol et al., 2021b). In general, there were significant improvements in the different parameters after the intervention was applied in the different studies.

A meta-analysis was carried out that included the objective variables of sleep efficiency, total sleep time, sleep latency period and time to wake up after the onset of sleep.

**Figure 3:**
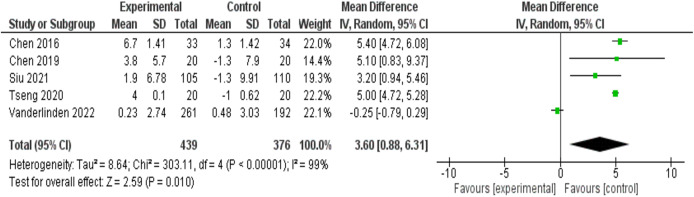
Forest and funnel plot of sleep efficiency measured with actigraphy. A positive mean difference favors the experimental (exercise) group. Between-study heterogeneity was substantial (I^2^ = 99%, τ^2^ = 8.64, *p* < 0.00001). Analyses were conducted in RevMan 5.4.

First, a mixed effects meta-analysis (Fig. 3) was performed with five studies (Chen et al., 2016, 2019; Tseng et al., 2020; Siu et al., 2021; Vanderlinden et al., 2022) on sleep efficiency, measured with actigraphy. The meta-analysis revealed an increase in standardized mean sleep efficiency (percentage) in favor of the intervention group of 3.60 (95% CI [0.88–6.31]), with I^2^ = 99%.

Additionally, a random effects meta-analysis (see Fig. S1 in the Supplemental Material) with five studies (Chen et al., 2016, 2019; Tseng et al., 2020; Siu et al., 2021; Vanderlinden et al., 2022) on total sleep time measured with actigraphy was carried out. The meta-analysis revealed an increase in the standardized mean total sleep time measured in minutes in favor of the intervention group of 1.47 (95% CI [−4.19 to 7.13]), with an I^2^ heterogeneity of 0%. The risk of publication bias of this meta-analysis is reflected in Fig. S1.

A random effects meta-analysis (see Fig. S2 in the Supplemental Material) was performed with four studies (Chen et al., 2016, 2019; Tseng et al., 2020; Siu et al., 2021; Seol et al., 2021a) on the sleep latency period measured with actigraphy. The meta-analysis revealed a decrease in the standardized mean sleep latency measured in minutes in favor of the intervention group of −3.86 (95% CI [−6.89 to −0.83]), with an I^2^ heterogeneity of 85%.

Finally, a random effects meta-analysis (see Fig. S3 in the Supplemental Material) was performed with five studies (Chen et al., 2016, 2019; Tseng et al., 2020; Siu et al., 2021; Vanderlinden et al., 2022) on the time to awaken after the onset of sleep (WASO). The meta-analysis revealed a decrease in the standardized mean measured in minutes in favor of the intervention group of −7.86 (95% CI [−18.08 to 2.35]), with an I^2^ heterogeneity of 85%.

### Subjective methods for measuring sleep quality

In addition to objective methods (actigraphy and polysomnography), different studies have used subjective methods to measure the quality and/or quantity of sleep, showing that after interventions, sleep is subjectively improved (Chen et al., 2016; Tseng et al., 2020; Siu et al., 2021; Seol et al., 2021a, 2021b).

The sleep diary was used in several studies to record the time spent in bed and getting up in the morning, to coordinate it with objective tools. The same parameters used with the objective methods were recorded for better analysis (Chen et al., 2016; Siu et al., 2021; Seol et al., 2021a).

The Pittsburgh Sleep Quality Questionnaire (PSQI) was used to measure self-reported sleep quality during the course of the intervention in several of the included studies (Tseng et al., 2020; Siu et al., 2021; Seol et al., 2021a; Vanderlinden et al., 2022).

Other similar scales have also been used, such as the Oguri-Shirakawa-Azumi Sleep Inventory (OSA-MA), a questionnaire specifically designed to evaluate the subjective quality of sleep the morning after sleeping at night (Seol et al., 2021b), and the Severity Index of the Insomnia (ISI) (Siu et al., 2021).

A random effects meta-analysis (see Fig. S4 in the Supplemental Material) was conducted with three studies (Barker et al., 2024; Chen et al., 2016; Siu et al., 2021) on sleep quality, which were evaluated *via* the PSQI questionnaire. The meta-analysis revealed a decrease in the standardized mean in favor of the intervention group of −2.34 (95% CI [−5.10 to 0.43]), with an I^2^ heterogeneity of 90%.

## Discussion

The results of this systematic review and meta-analysis show the efficacy of implementing physical activity programs to improve poor sleep quality or insomnia in non-hospitalized elderly people, specifically through the use of objective tools. These results coincide with current scientific research that promotes the implementation of non-pharmacological, sustainable and adaptable interventions in community contexts (Gu & Lee, 2023; Vásquez-Carrasco et al., 2025).

Despite the heterogeneity in the activities, in general, the majority of physical exercises have shown s benefits, with a relationship between physical exercise and improvements in sleep quality 45–50. However, one of the studies did not show statistically significant results or improvements in sleep quality, which is probably due to the low frequency of the intervention (one session per week for 12 weeks) and limited adherence by the participants (Vanderlinden et al., 2022). Other studies with fewer sessions also reported no differences (Kovacevic et al., 2018), which highlights that sports interventions only generate positive effects on sleep when they are maintained for at least 8 weeks. In contrast, the study by Tseng et al. (2020) stands out for its effectiveness, since with an intervention of 12 weeks and 3 weekly sessions, it obtained the best results in improving the quality of sleep. Studies with higher session frequency and better adherence tended to report larger improvements; however, we did not perform formal moderator analyses, and these patterns should be considered hypothesis-generating, not causal.

Gender differences in sleep quality are consistently reported among older adults and by various studies, with women exhibiting worse subjective and objective sleep parameters than men do, finding it more difficult to fall asleep and suffer more awakenings. These disparities are multifactorial. Biologically, post-menopausal decreases in estrogen and progesterone contribute to vasomotor instability, thermoregulation changes, and alterations in melatonin secretion that impair sleep initiation and maintenance. Women also present a greater incidence of psychiatric pathology with high rates of depression and anxiety, which can mediate insomnia symptoms. Socially, factors such as living alone and having a lower income at an older age further exacerbate sleep disruption. Recognizing these biological and psychosocial determinants is essential for tailoring exercise and other non-pharmacological interventions by sex (Ancoli-Israel & Martin, 2023; Salis et al., 2024; Chu, Oh & Lee, 2022; Baker & Driver, 2022).

Therefore, it is necessary for the interventions to consider gender differences to enhance their comprehensiveness and effectiveness (Kohanmoo et al., 2024).

In addition, from a chronobiological perspective, aging is associated with a progressive desynchronization of the suprachiasmatic nucleus (SCN), the central pacemaker that regulates the circadian rhythms of sleep, metabolism, and cognition. Physical activity, especially when performed outdoors with exposure to natural light, acts as a potent zeitgeber that reinforces circadian rhythmicity by increasing clock gene expression and increasing rhythm amplitude. Experimental models have demonstrated that regular exercise promotes the expression of PER and BMAL1 genes in SCN neurons, stabilizing circadian oscillations and improving sleep–wake synchronization. In older adults, higher levels of physical activity are correlated with greater circadian amplitude and better sleep consolidation. Therefore, timed physical activity, particularly morning or daylight outdoor exercise, may synergize with natural light exposure to optimize circadian entrainment, contributing not only to improved sleep quality but also to cognitive preservation and overall healthy aging. Integrating these mechanisms into personalized exercise prescriptions may enhance therapeutic strategies for circadian health in the elderly (Hood & Amir, 2023; Tanaka, Sagayama & Shimizu, 2023; Golombek & Rosenstein, 2010).

An additional, yet relatively unexplored, factor is the timing of exercise in relation to individual chronotype. Differences in chronotype affect the synchronization of circadian rhythms and consequently modify the influence of physical activity on sleep quality. Exercising in the morning tends to advance the circadian phase and promote nocturnal melatonin production, whereas exercising late at night can delay this phase and negatively affect sleep efficiency in some individuals. Therefore, tailoring exercise schedules to individuals’ natural circadian preferences could improve the effectiveness of interventions aimed at improving sleep and overall well-being in older adults. Recent research evidence indicates that physical activity performed in the morning or during the day is associated with improved actigraphic sleep efficiency and more stable circadian patterns than in evening exercise, especially in individuals with later chronotypes. Therefore, integrating chronotype assessment into exercise program planning may encourage greater adherence and optimize therapeutic outcomes in this population (Vitale, Bonato & La Torre, 2024; Seol et al., 2021a; Buxton & Wright, 2023).

One potential line of future research is to consider how genetic differences between individuals might influence the design of physical activity programs aimed at improving sleep and circadian health in older adults. Recent genome-wide association studies have shown that certain genetic variants related to circadian and sleep regulation can influence aspects such as sleep duration, chronotype, and other health outcomes (Lane et al., 2023). Some polymorphisms, such as the CLOCK 3111 T/C variant, have even been linked to changes in daily activity rhythms, suggesting that genetic factors may influence individual behavior patterns (Miyazaki et al., 2022). Identifying these genetic profiles and combining them with various sleep assessment tools, such as actigraphy, polysomnography, or standardized questionnaires, could allow for the personalization of exercise programs. This would allow practitioners to adjust not only the type and timing of activities but also align them with the biological and genetic characteristics of each individual. This approach could improve the adherence, accuracy, and effectiveness of interventions in diverse populations of older adults (Vitale, Bonato & La Torre, 2024; Lane et al., 2023; Miyazaki et al., 2022).

The use of objective methods (polysomnography and actigraphy) is a key tool in the present systematic review for the evaluation of sleep. Unlike subjective methods such as scales or questionnaires, objective methods allow continuous monitoring for several days and even weeks and are very useful tools since the subjective perception of sleep may not be accurate due to a lack of awareness of sleep or cognitive alterations in older adults. While actigraphy provides valuable and objective information about sleep parameters over extended periods, it is not equivalent to polysomnography, which remains the gold standard for assessing sleep architecture, respiratory events, and electrophysiological parameters. However, its feasibility, lower cost, and ability to record habitual sleep in natural environments make it a viable alternative for large-scale, longitudinal studies with older adults (Ancoli-Israel & Martin, 2023; Chen et al., 2016, 2019; Tseng et al., 2020; Siu et al., 2021; Seol et al., 2021a; Vanderlinden et al., 2022; Neikrug, 2023; Haghayegh et al., 2019).

However, although physical activity programs have proven to be effective, different types of non-pharmacological methods are currently being investigated to treat insomnia or poor quality of sleep, including cognitive behavioral therapy for insomnia (McCrae et al., 2018; Dzierzewski et al., 2019), combined programs of physical exercise with cognitive therapy (Kline et al., 2022) or acupuncture (Ferreira et al., 2022), virtual reality (Chitra & Eremita, 2023; Cinalioglu et al., 2023) or music therapy (Chen et al., 2021). Cognitive behavioral therapy for insomnia (CBT-I) is currently considered the gold standard non-pharmacological treatment for chronic insomnia, and international guidelines recommend it as a first-line therapy. However, its accessibility can be limited by the need for trained therapists, the cost of sessions, and lower treatment adherence among older adults. In this context, physical-activity programs can serve as an alternative or complementary approach that is cost-effective, feasible in community settings, and capable of addressing multiple health dimensions, including mood and circadian regulation (Riemann et al., 2023; Qaseem et al., 2016).

Likewise, physical activity, in addition to improving the quality of sleep, provides significant benefits in multiple dimensions of health in old age. It acts as a protective factor against cardiovascular and metabolic diseases and certain types of cancer, and is associated with a delay in the appearance of cognitive deterioration, as well as with better mental health, higher quality of life and general well-being (Langhammer, Bergland & Rydwik, 2018; Pinheiro et al., 2022). A relevant aspect is the social component of these programs, since their group format contributes to reducing loneliness and depressive symptoms, especially in older adults who live alone, by facilitating the creation of social support networks (Sebastião & Mirda, 2021; Zimmer et al., 2023). The included studies, highlighted how participation in these interventions facilitates compliance with the physical activity recommendations (150 min per week) and improves adherence when performed in groups. Likewise, regular physical exercise has positive effects on cardiovascular function (Tseng et al., 2020; Seol et al., 2021a; Vanderlinden et al., 2022).

However, pharmacological treatment is still the most widely used option, because of its short-term efficacy and because it does not require learning or training on the part of the patient. However, there is evidence of its limitations, such as the appearance of dependence, tolerance, and adverse side effects (Rios et al., 2019; Carrillo-Mora et al., 2018). Benzodiazepines are among the most widely used drugs; however, their use is not recommended for more than 4 weeks, and they should be withdrawn progressively to avoid rebound effects (Álvarez-Ruiz de Larrinaga et al., 2022).

Faced with these limitations, physical exercise is presented as a plausible and cost-effective alternative. The evidence shows benefits in the quality of sleep, and its implementation can be carried out in a structured way in community settings or health centers, with minimal investment, taking advantage of existing human resources (such as physiotherapists or nursing staff). This allows a comprehensive and interdisciplinary approach, as well as continuous monitoring by health professionals (Lin et al., 2020; Rocha et al., 2024).

In addition, in the studies analyzed, a decrease in the consumption of hypnotics was observed after the application of interventions based on physical exercise, which reinforced their practical usefulness and acceptance among the participants (Seol et al., 2021a).

Although there is currently no specific physical programme focused exclusively on sleep, physical exercise is included in initiatives such as the European project Health-Enhancing Physical Activity (HEPA) of the WHO-Europe, which promotes healthy aging through physical activity. In this context, the included studies highlight the use of the Lekker Actief program, implemented in Belgium, which promotes a healthy lifestyle in older adults through exercise, social interaction and a balanced diet (Vanderlinden et al., 2022).

### Limitations

This systematic review demonstrated how effective physical activity programs are in improving the quality of sleep. However, the heterogeneity of physical exercise programs should be considered in terms of the type of exercise, duration, and frequency. The very high between-study heterogeneity (*e.g*., sleep efficiency I^2^ ≈ 99%) is unsurprising given the clinical, intervention, measurement, and methodological diversity across trials. Under these conditions, a random-effects meta-analysis estimates a mean of potentially different true effects, so the pooled value should be interpreted as reflecting direction and average magnitude, not a single expected effect for all contexts.

A futher limitation concerns that most of the included studies had relatively small sample sizes, often fewer than 100 participants per group, and were conducted in specific geographic or community settings. These factors may limit the generalizability of the findings to broader older adult populations with different sociodemographic or health characteristics. Furthermore, variability in intervention duration and exercise type further limits external validity. Therefore, future studies should include larger, multicenter, randomized controlled trials with diverse populations to confirm the reproducibility and scalability of these results.

Another limitation would be that the participants were mostly women, and it is known that gender may have a different response with respect to exercise; Thus, it is necessary to conduct sex-disaggregated analyses in future studies. It would also be beneficial for future research to compare or combine the effectiveness of physical exercise with that of other non-pharmacological therapies, such as cognitive-behavioral interventions.

The strengths of this systematic review, include the choice of high-quality randomized clinical trials and the use of both objective and subjective tools.

## Conclusions

Physical activity programs have proven to be safe and effective in improving the quality of sleep in non-hospitalized elderly people; specifically, they are practiced frequently. They are able to improve the latency period, efficiency, total time and number of awakenings after the onset of sleep, as measured by objective and subjective tools. Furthermore, they are interventions with comprehensive positive benefits for people and are a plausible alternative in terms of cost effectiveness compared with non-pharmacological interventions. The implementation of these interventions in health systems and community settings should be promoted, leveraging existing personnel and material resources to enable structured interventions at low additional cost since it is an effective and complete alternatives in the care of the elderly population that reduce pathologies and comorbidities and improve sleep-related outcomes in older adults.

## Supplemental Information

10.7717/peerj.20764/supp-1 Supplemental Information 1 PRISMA checklist.

10.7717/peerj.20764/supp-2 Supplemental Information 2 Supplemental Material.

10.7717/peerj.20764/supp-3 Supplemental Information 3 Raw data. Raw data extracted from included studies, used for meta-analysis. Includes pre-post means and standard deviations for sleep latency, efficiency, total sleep time, WASO, and PSQI in intervention and control groups.

10.7717/peerj.20764/supp-4 Supplemental Information 4 Rationale.

## Additional Information and Declarations

### Data Availability

The following information was supplied regarding data availability:

The raw data are in the Supplemental File.
