# Supplementary material for: Physical exercise programmes to improve insomnia or poor sleep quality in non-hospitalised elderly people: a systematic review and meta-analysis
Source: PeerJ. 2026 Feb 16;14:e20764. doi: 10.7717/peerj.20764 (PMC12919316; doi:10.7717/peerj.20764)
Supplement: Supplemental Information 2 [file peerj-14-20764-s002.docx]

**Supplemental Material (S)**

1. **Figure S1:** Forest plot of total sleep time measured with actigraphy


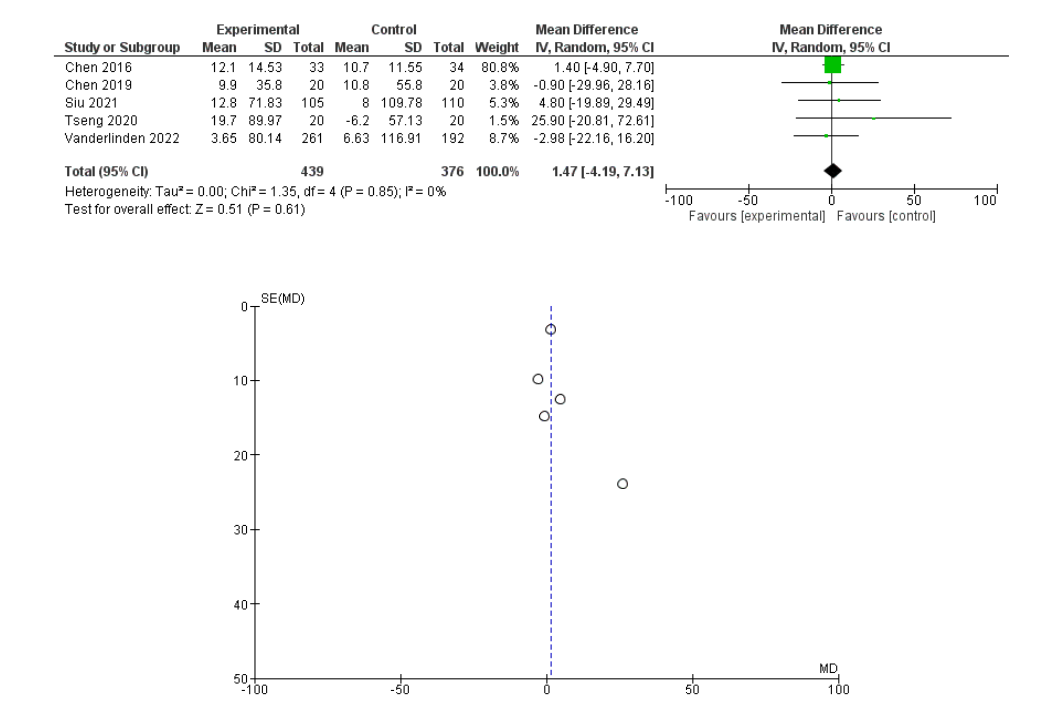


1. **Figure S2:** Forest plot of the sleep latency period measured with actigraphy.


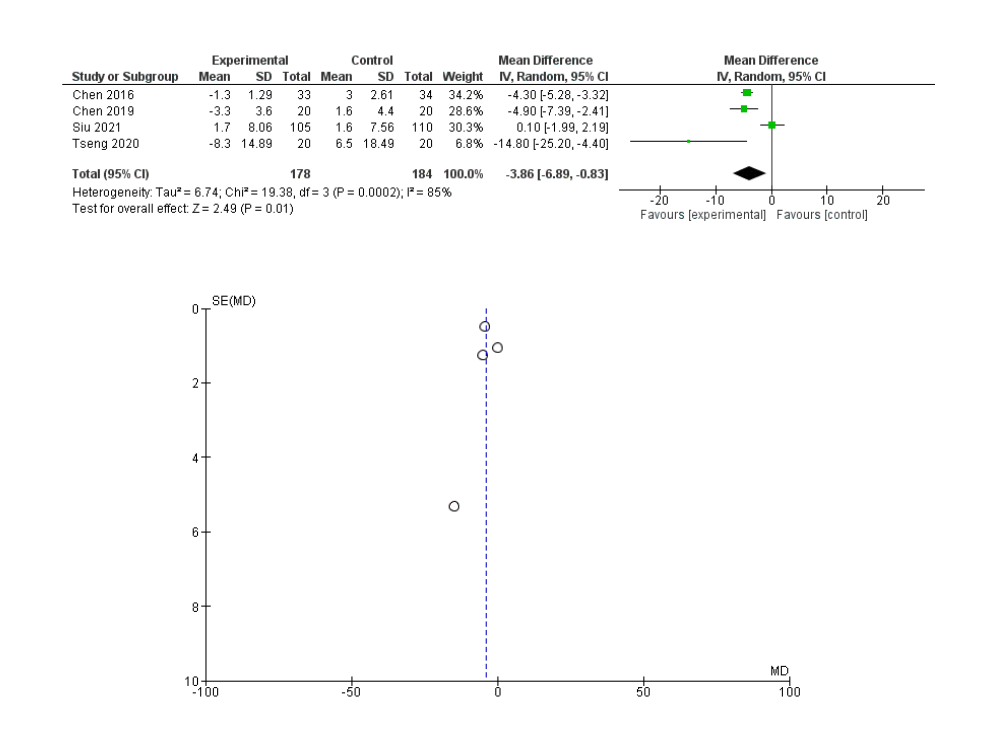


1. **Figure S3:** Forest plot of the time to awakening after the onset of sleep measured with actigraphy.


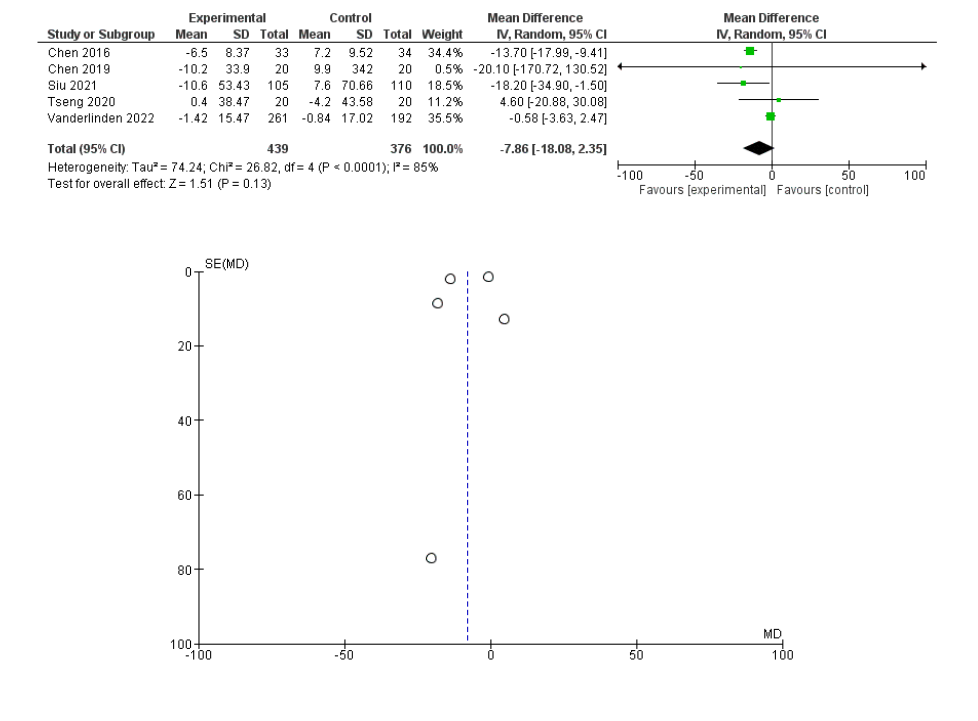


1. **Figure S4:** Forest plot for the evaluation of the quality of sleep with the PSQI.


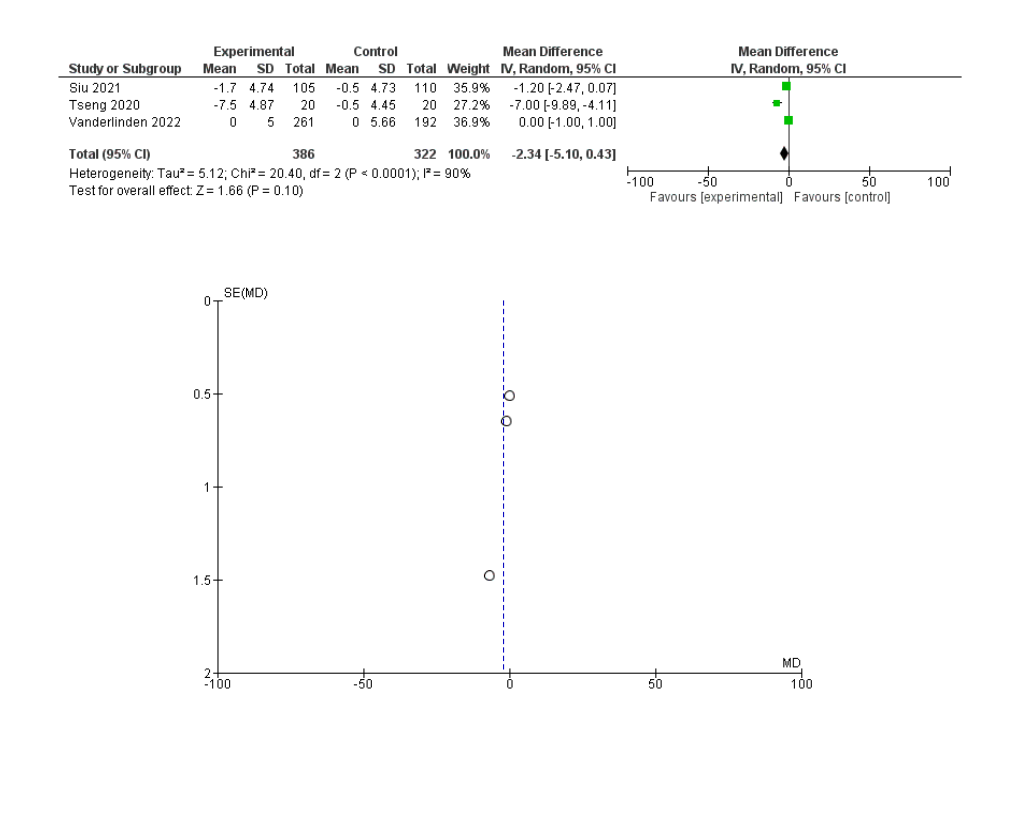


1. **Table S1:** Application of the Joanna Briggs Guide for the pretest posttest study included in the review.

|  | Vanderlinden J et al. (51). |
| --- | --- |
| 1. Is it clear from the study what is the "cause" and what is the effect? | Yes |
| 1. Were the participants included in any of the comparisons similar? | Yes |
| 1. Were participants included in any comparison receiving similar treatment care, other than the exposure or intervention of interest? | No |
| 1. Was there a control group? | Yes |
| 1. Were there multiple   outcome measures before and after the intervention exposure? | Yes |
| 1. Was the follow-up and, if not, were the differences between the groups in terms of follow-up adequately described and analyzed? | Yes |
| 1. Were the outcomes of the participants included in any comparison measured in the same way? | Yes |
| 1. Were the results measured reliably? | Yes |
| 1. Was an adequate statistical analysis used? | Yes |
