# Supplementary material for: Physical exercise programmes to improve insomnia or poor sleep quality in non-hospitalised elderly people: a systematic review and meta-analysis
Source: PeerJ. 2026 Feb 16;14:e20764. doi: 10.7717/peerj.20764 (PMC12919316; doi:10.7717/peerj.20764)
Supplement: Supplemental Information 4 [file peerj-14-20764-s004.docx]

*Audience justification*: Physical exercise programmes to improve insomnia or poor sleep quality in non-hospitalised elderly people: a systematic review and meta-analysis.

This systematic review and meta-analysis is intended for the following audiences:

1. **Healthcare professionals**, especially those involved in geriatric care, such as nurses, physiotherapists, general practitioners, geriatricians, and sleep specialists. The findings provide evidence-based guidance on non-pharmacological interventions, specifically physical activity programmes, that can be implemented to improve sleep quality in older adults.
2. **Clinical researchers and academics** in the fields of sleep medicine, aging, and rehabilitation who are interested in the effectiveness of physical exercise as a therapeutic tool to manage insomnia and poor sleep quality in elderly populations.
3. **Public health professionals and policymakers** seeking cost-effective, accessible, and safe strategies to promote healthy aging and reduce the use of sleep medication among the elderly. This evidence can inform the design of community-based interventions and support policies promoting physical activity in older populations.
4. **Students and educators** in health sciences (e.g., nursing, physiotherapy, gerontology, public health) who can use the synthesized evidence to understand the role of physical activity in improving sleep and overall well-being in the aging population.

By targeting these groups, the review aims to support clinical decision-making, encourage multidisciplinary approaches, and contribute to the implementation of practical and sustainable health interventions for older adults.
